# Effectiveness of nurse-led intervention on eating triggers for weight control: a quasi-experimental study

**DOI:** 10.3389/fpubh.2026.1749283

**Published:** 2026-04-29

**Authors:** Mohammed Faris Abdulghani, Sadeq Al-Fayyadh, Tahsein Muhsin Hussein

**Affiliations:** 1 College of Nursing, Nineveh University, Mosul, Iraq; 2 College of Nursing, University of Baghdad, Baghdad, Iraq; 3 College of Nursing, University of Mosul, Mosul, Iraq

**Keywords:** eating triggers, nurse-led intervention, obesity, quasi-experimental study, weight control

## Abstract

**Background:**

Overweight and obesity remain significant public health challenges requiring urgent solutions. Despite progress, these conditions continue to rise globally, underscoring the need for more robust and innovative approaches. Therefore, this non-randomized quasi-experimental study aimed to evaluate the effectiveness of a nurse-led intervention on eating triggers for weight control among adults with obesity.

**Methods:**

This non-randomized quasi-experimental study included 128 participants recruited through convenience sampling and allocated into an intervention group (*n* = 64) and a control group (*n* = 64). The sample consisted of 78 women (60.9%) and 50 men (39.1%). The intervention group received a tailored nurse-led educational and behavioral program over a three-month period, while the control group received standard recommendations. Participants were assessed using a food triggers questionnaire before and after the intervention. The primary outcome was change in eating trigger scores and the secondary outcome was change in Body Mass Index (BMI). Data were analyzed using independent and paired *t*-tests and analysis of covariance (ANCOVA) to control for potential confounding variables.

**Results:**

The intervention group demonstrated a significant reduction in total eating trigger scores compared with the control group (Mean difference = −11.890, *P* < 0.001, partial η^2^ = 0.469). Additionally, the results showed the BMI decreased significantly in the intervention group compared with the control group (Mean difference = −8.005, *P* < 0.001), partial η^2^ = 0.713.

**Conclusion:**

This study demonstrates that a nurse-led intervention was effective in reducing eating trigger scores and improving BMI among adults with obesity following a three-month intervention period. Further studies with extended follow-up periods are recommended to evaluate the long-term sustainability of these outcomes.

## 1 Introduction

Overweight and obesity are major global public health concerns characterized by abnormal or excessive fat accumulation that increases the risk of adverse health outcomes (1, 2). Body Mass Index (BMI) is commonly used as a practical indicator to classify weight status, with overweight defined as BMI ≥ 25 kg/m^2^ and obesity as BMI ≥ 30 kg/m^2^ (2). However, recent conceptual frameworks emphasize that obesity should be understood as a complex chronic disease characterized not only by excess body fat but also by its metabolic, functional, and psychosocial consequences. According to the updated definition proposed by Rubino et al., clinical obesity is defined as excess adiposity that impairs health and should be considered a chronic disease, indicating that BMI alone may not fully capture the complexity of the condition(3). The global prevalence of overweight and obesity has risen substantially over recent decades, with more than one billion individuals currently affected worldwide (4). These conditions are strongly associated with increased risk of chronic diseases, including cardiovascular disease, type 2 diabetes, and several types of cancer, contributing significantly to morbidity and premature mortality (5). With a steady increase in the number of individuals with overweight and those with obesity, there is a growing public health concern about body size and lifestyle diseases (6). Therefore, reducing the prevalence of overweight and obesity is a key public health priority (7).

Obesity is widely recognized as a multifactorial chronic disease influenced by complex interactions among biological, environmental, psychological, and social determinants. While excessive energy intake and overeating behaviors contribute to positive energy balance and weight gain, these behaviors represent only one component of a broader set of factors that influence obesity development (8). Therefore, understanding the triggers that lead to eating in everyday life is crucial for developing interventions that promote healthy eating and prevent overeating (9). What drives individuals to eat? While physiological factors play a significant role in human eating behavior, various psychological factors influence eating habits at any meal (9). Previous research has identified several key psychological drivers, including emotional eating (10), stress-related eating (11), impulsivity, reward sensitivity (12), and maladaptive cognitive patterns related to food (13, 14). Emotional distress, such as anxiety, depression, and negative mood states, has been shown to increase the likelihood of consuming high-calorie and highly palatable foods as a coping mechanism (10). Stress exposure can also alter appetite regulation through neuroendocrine pathways, particularly through cortisol-mediated responses (11). Additionally, cognitive factors such as dysfunctional beliefs about food, reduced self-control, and attentional bias toward food cues may increase susceptibility to overeating (13). Social influences, including peer pressure, cultural norms, and environmental food availability, further shape eating patterns and contribute to unhealthy dietary behaviors (14).

Nurses play a crucial role in chronic disease management, acting as frontline healthcare providers. Their responsibilities include patient education, ongoing monitoring, and care coordination, all aimed at improving patient outcomes. Nurses often guide lifestyle interventions, ensure medication adherence, and emotionally support individuals with chronic conditions. Their holistic approach addresses not only the physical aspects of the disease but also the psychological and social dimensions, fostering a patient-centred care model. The collaborative efforts of nurses significantly enhance the overall wellbeing and quality of life for those living with chronic diseases (15, 16). Nursing-led interventions are based on a care delivery model that incorporates assessment, evaluation, education, counselling, treatment, and other procedures using a comprehensive nurse-patient (family) approach. The nursing professional works independently or in interdisciplinary teams (17).

Lifestyle modification interventions, particularly those led by nurses, have proven effective in lowering cardiovascular risk scores across various populations (18, 19). Lifestyle intervention research has extensively examined the outcomes of nurse-led diet and exercise interventions, demonstrating improvements in several health indicators, including reductions in body mass index, waist circumference, cardiovascular risk factors, and improvements in dietary behaviors and physical activity levels (20–22). Additionally, a systematic review of 18 studies evaluating nurse-led interventions found that diet and exercise were more effective in preventing childhood and adolescent overweight and obesity. (23).

While existing research has demonstrated the effectiveness of nurse-led interventions in managing obesity and reducing health risks, overweight and obesity remain significant public health challenges that require urgent and effective solutions. Despite progress, these conditions continue to rise globally, underscoring the need for more robust and innovative approaches. Additionally, according to the researchers' knowledge, there is a noticeable gap in literature. However, limited research has specifically examined interventions designed to modify eating triggers or stimuli that influence eating behavior in the context of weight management. In particular, few studies have assessed whether targeted behavioral interventions can alter eating trigger patterns measured through structured assessment tools. This gap highlights a fundamental need to strengthen further and refine existing strategies. Addressing these triggers through targeted interventions could enhance the effectiveness of weight loss programs, making them more comprehensive and capable of producing sustainable results. Therefore, the present study aimed to evaluate the effectiveness of a nurse-led intervention on eating triggers for weight control in adults with obesity using a non-randomized quasi-experimental design. It was hypothesized that a tailored nurse-led educational and behavioral intervention would significantly reduce eating trigger scores across psychological, social, situational, cognitive, and physiological domains and lead to improved weight-related outcomes, including reduced body mass index, compared with standard lifestyle counseling provided during routine outpatient care.

## 2 Methods

### 2.1 Study design and setting

The study employed a non-randomized quasi-experimental design. This study was reported in accordance with the Transparent Reporting of Evaluations with Nonrandomized Designs (TREND) statement. It was conducted in the Alkindi School of Medicine under the Ministry of Higher Education and Scientific Research and the Nutrition Research Center under the official umbrella of the Iraqi Health Ministry.

### 2.2 Sample size and sampling

A purposive non-probability sample of 128 first-time clients was recruited from individuals attending the Obesity Control and Research Unit at the University of Baghdad, Al-Kindi School of Medicine, and the Nutrition Research Center under the Iraqi Ministry of Health. Eligible participants were individuals seeking obesity management services, able to communicate effectively, and willing to participate in the study. The inclusion criteria consisted of participants with no prior history of consultation, follow-up, or treatment at the Obesity Control and Research Unit within the previous 12 months and were attending the unit for the first time during the study recruitment period, had a Body Mass Index (BMI) ≥ 30 kg/m^2^, and voluntarily agreed to participate by signing the informed consent form. Exclusion criteria included individuals with conditions that severely impaired cognitive function and the ability to understand or consent to the study, individuals under 18 years of age, and participants with critical illnesses.

**Figure 1:**
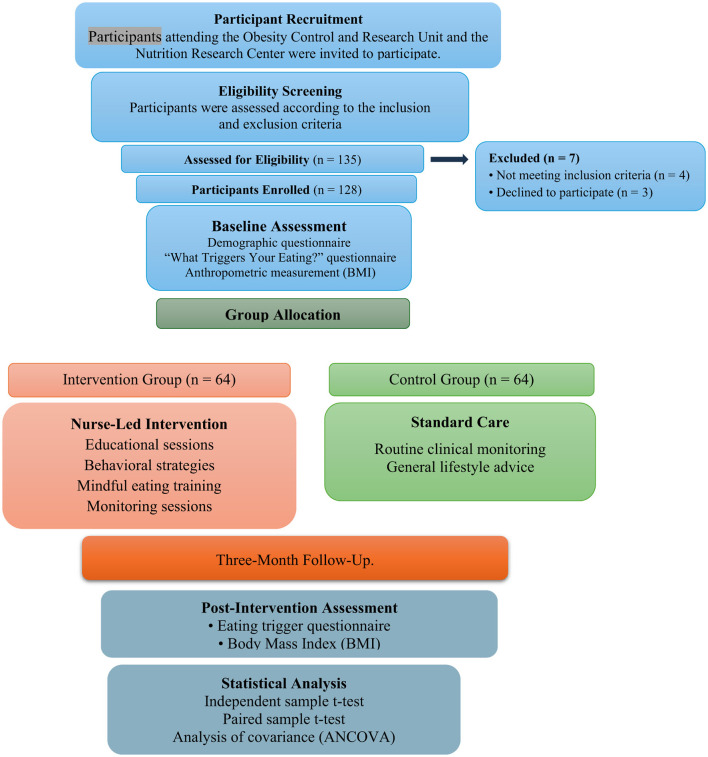
Participant selection flowchart.

The required sample size was calculated using G^*^Power software (version 3.1) based on Student's *t*-test analysis, assuming an anticipated effect size (Cohen's d) of 0.5, a statistical power of 0.80, and a significance level of 0.05 (24) (Figure 1).

Participants were allocated to intervention and control groups using convenience allocation based on participant availability and scheduling of the intervention program, as randomization was not feasible within the quasi-experimental design. No randomization procedure was applied due to the quasi-experimental design of the study.

### 2.3 Data collection tools

A comprehensive questionnaire was utilized to gather data, including two primary subsections: the socio-demographic, cultural-religious, and disease history information registration form and the What Triggers Your Eating? Questionnaire.

Socio-demographic, cultural-religious, and disease history information: This form involved several elements, including gender, age categorical, age in years, education, employment, residency, marital status, income, religious rules, legislation, medical history, and BMI.

What Triggers Your Eating? Questionnaire: The study used the certified Arabic version of the “*What Triggers Your Eating?” questionnaire*, originally developed by Nash (1997) in the book “*The New Maximize Your Body Potential”* (25). The questionnaire comprises 20 items designed to assess eating triggers across five domains: emotional, social, situational, cognitive (thinking-related), and physiological.

Emotional triggers include both negative emotional states, such as stress, anxiety, sadness, loneliness, and boredom, as well as positive emotional situations, such as celebrations or reward-related eating. Social triggers involve eating during social gatherings, peer influence, or family-related food practices. Situational triggers refer to environmental cues such as food availability, exposure to food advertisements, or specific eating routines. Cognitive triggers include thoughts and beliefs related to food, dieting, or self-control. Physiological triggers assess hunger, fatigue, and physical discomfort associated with eating behavior.

Each item is rated on a 10-point Likert scale ranging from 1 (”very unlikely to start eating”) to 10 ("very likely to start eating”). Higher scores indicate a stronger likelihood of eating in response to a particular trigger.

Content validity of the Arabic version was evaluated by a panel of experts in nursing, nutrition, and behavioral sciences to ensure the items were clear, relevant, and adequate for the study objectives. Minor linguistic adjustments were made in response to expert feedback to improve clarity and cultural appropriateness. The instrument demonstrated good internal consistency reliability with a Cronbach's alpha of 0.90, while criterion validity showed a strong correlation (*r* = 0.803, *p* < 0.001).

Dominant eating triggers were identified by calculating the total score within each trigger domain. The domain with the highest score was considered the participant's predominant eating trigger.

**Body Mass Index:** Body weight and height were measured objectively by trained research staff using standardized procedures. Body weight was measured using a calibrated digital scale (Seca^®^, Germany), with participants wearing light clothing and no shoes. The scale was regularly calibrated according to manufacturer guidelines. Height was measured using a stadiometer with participants standing upright without footwear. Body mass index (BMI) was calculated using the standard formula: weight in kilograms divided by height in meters squared (kg/m^2^).

BMI classifications were defined according to the World Health Organization criteria as follows: normal weight (18.5–24.9 kg/m^2^), overweight (25.0–29.9 kg/m^2^), obesity class I (30.0–34.9 kg/m^2^), obesity class II (35.0–39.9 kg/m^2^), and obesity class III (≥ 40.0 kg/m^2^).

### 2.4 Data collection

After obtaining approval from the relevant institutional authorities, data collection was initiated at the Obesity Control and Research Unit. Eligible participants were informed about the study objectives and procedures, and written informed consent was obtained prior to participation. Participants then completed baseline assessments using the “What Triggers Your Eating?” questionnaire.

The intervention was designed as a tailored nurse-led program aimed at addressing each participant's predominant eating triggers. Initially, participants were assessed using the questionnaire to identify their primary eating triggers, which were categorized into five domains: emotional, social, situational, cognitive (thinking-related), and physiological triggers. Based on the assessment results, individualized intervention plans were developed to target participants' dominant triggers.

The intervention was delivered by registered nurses with an average of approximately 6–8 years of clinical experience in obesity management and lifestyle counseling. Educational sessions were conducted weekly and lasted approximately 45–60 min. Group sessions included 5–8 participants to facilitate interaction while maintaining individual engagement. Individual sessions were provided when participants required additional support. All sessions were conducted in private consultation rooms at the Obesity Control and Research Unit to ensure confidentiality and a supportive environment. Participants were assigned identification codes, and all personal information and shared discussions were treated as confidential and accessible only to the research team.

Trigger-specific educational sessions focused on increasing awareness and providing coping strategies tailored to each trigger category. Emotional trigger sessions addressed emotion regulation, mindfulness techniques, and identification of emotional states associated with eating behaviors. Social trigger sessions focused on managing social pressure, assertiveness training, and strategies for navigating social gatherings involving food. Situational trigger sessions emphasized managing environmental cues and modifying routines that promote overeating. Cognitive trigger sessions addressed dysfunctional beliefs and thought patterns related to eating through cognitive restructuring strategies. Physiological trigger sessions provided nutrition education focusing on recognizing hunger and satiety signals and distinguishing physiological hunger from psychological or emotional eating.

In addition to educational sessions, participants received individualized behavioral therapy sessions conducted by trained nurses with experience in behavioral counseling and supervised by a behavioral health specialist, using cognitive-behavioral therapy techniques. These sessions lasted 30–45 min and were conducted weekly or biweekly depending on participant needs. Behavioral therapy focused on increasing awareness of eating behaviors, challenging maladaptive thoughts, exposure to trigger situations in controlled settings, problem-solving skills, and relapse prevention strategies.

Peer support was provided through structured support group meetings facilitated by healthcare professionals. These meetings were conducted twice monthly and included guided group discussions, role-playing exercises to practice responses to eating triggers, and peer mentoring activities to encourage experience sharing and mutual support among participants.

During the intervention period, structured monitoring sessions were conducted to support participant progress and reinforce behavioral strategies. These sessions were scheduled at a minimum frequency of once every 2 weeks and up to once per week, depending on individual participant needs. Each session lasted approximately 30 min and included reassessment of eating triggers using the same standardized questionnaire, provision of individualized feedback, and adjustment of intervention strategies based on participant progress. All sessions were conducted according to a standardized protocol and documented using structured checklists to ensure consistency and intervention fidelity.

Participants in the control group received standard care routinely provided by the treatment unit, including routine clinical monitoring, medical consultation, and general lifestyle advice on weight management. However, the control group did not receive the structured trigger-based behavioral intervention provided to the intervention group.

### 2.5 Ethics approval and consent to participate

The Institutional Review Board (IRB) at the University of Baghdad's College of Nursing approved the study after the study proposal was submitted. All the information provided during the research project was documented so that their identities remained confidential. Only the principal investigator can access the collected data, which is securely stored. Additionally, participants were informed that they could share only what they felt comfortable disclosing and that they had the right to withdraw from the study at any time at their discretion. All informed consent forms were signed by the participants or their legal guardians. They were made aware that participation was entirely voluntary and that they had the right to read, discuss, and ask questions about the study protocol and the benefits and risks of participation with the researcher.

### 2.6 Data analysis

To minimize the influence of potential confounding variables, participants in both intervention and control groups received general standardized recommendations regarding balanced dietary habits and physical activity consistent with national health guidelines. Participants were advised to maintain their usual physical activity levels throughout the study period unless otherwise medically indicated. Additionally, baseline characteristics, including dietary habits and health history, were collected through questionnaires. Participants were asked to report any major changes in dietary habits or physical activity during monitoring sessions to monitor behavioral consistency and reduce potential confounding effects.

Data were analyzed using SPSS software version 25. Descriptive statistics, including mean, standard deviation, frequency, and percentage, were used to summarize participants' demographic and baseline clinical characteristics. To evaluate within-group changes across time, paired sample *t*-tests (or Wilcoxon signed-rank tests for non-normally distributed variables) were conducted to compare baseline and post-intervention outcomes in each group. Between-group differences were assessed using independent sample *t*-tests (or Mann–Whitney *U*-tests for non-parametric data) to compare mean changes in outcome variables between the intervention and control groups.

For repeated outcome measurements collected across multiple time points, repeated-measures analysis of variance (ANOVA) or mixed-effects models were performed to examine time effects, group effects, and interaction effects between time and group. Where significant interactions were identified, *post hoc* analyses with Bonferroni correction were applied. To control for potential confounding variables, analyses were adjusted for baseline demographic and clinical characteristics, including age, sex, baseline Body Mass Index (BMI), physical activity level, and dietary patterns. Covariate adjustments were performed using analysis of covariance (ANCOVA) or multivariable regression models, depending on the outcome variable. Assumptions of normality and homogeneity of variance were evaluated using the Shapiro–Wilk test and Levene's test, respectively. A *p*-value of less than 0.05 was considered statistically significant.

## 3 Results

The mean ages of participants in the intervention and control groups were 32.64 ± 10.56 and 35.61 **±** 11.05 years, respectively. The majority of participants in both groups were male. 71.9% of the participants in the control group and 43.8% in the intervention group were married. There was no significant difference between the two groups regarding age, gender, religious rules, income, and history of chronic disease (*P* > 0.05). There was a significant difference between the two groups regarding marital status, education level, job, and living place (*P* < 0.05) (Table 1).

**Table 1:** Comparison of the participants' characteristics between the two groups.

Groupvariable	Intervention (*n* = 64)		Control (*n* = 64)		Statistical test	*P*-value
	Mean	SD	Mean	SD		
Age (yr.)	32.64	10.56	35.61	11.05	*t* = 1.554	0.123
	n	%	n	%		
Age (yr.)						
18–28	28	43.8	21	32.8	χ^2^ = 1.730	0.630
29–39	18	28.1	20	31.3		
40–49	13	20.3	17	26.6		
50–59	5	7.8	6	9.4		
Gender						
Male	41	64.1	38	59.4	χ^2^ = 0.298	0.685
Female	23	35.9	26	40.6		
Marital status						
Married	28	43.8	46	71.9	χ^2^ = 10.433	0.005
Single	29	45.3	15	23.4		
Divorce/widower	7	10.9	3	4.7		
Education level						
Able to read and write	6	9.4	2	3.1	χ^2^ = 9.922	0.042
Primary school	8	12.5	1	1.6		
Secondary school	8	12.5	6	9.4		
High school	22	34.4	25	39.1		
> High school	20	31.2	30	46.9		
Job						
Employed	36	63.2	21	34.4	χ^2^ = 9.741	0.002
Unemployed	21	36.8	40	65.6		
Living place						
Rural	1	1.6	8	12.5	χ^2^ = 5.856	0.016
Urban	63	98.4	56	87.5		
Income						
Sufficient	28	43.8	17	26.6	χ^2^ = 5.132	0.077
Barely sufficient	24	37.4	36	56.3		
Not sufficient	12	18.8	11	17.2		
Religious rules						
Yes	42	65.6	36	56.3	χ^2^ = 4.562	0.102
No	7	10.9	3	4.7		
Don't know	15	23.5	25	39.0		
History of chronic disease						
Hypertension	9	14.1	9	14.1	χ^2^ = 3.288	0.349
Angina pectoris	5	7.8	1	1.6		
Diabetes	12	18.8	10	15.6		
No	38	59.3	44	68.7		

SD, standard deviation; t, independent *t*-test; χ^2^, Chi square test.

The total score of eating triggers and all trigger dimensions decreased significantly in the intervention group compared with the control group following the intervention (*p* < 0.05). After controlling for potential confounding variables, including marital status, education level, occupation, place of residence, and baseline eating trigger scores, ANCOVA analysis demonstrated a statistically significant reduction in the overall eating trigger score in the intervention group compared with the control group (mean difference = −11.890, *p* < 0.001, effect size = 0.469). Adjusted analyses also demonstrated significant reductions across all trigger dimensions. The largest effect was observed in emotional eating triggers (mean difference = −14.834, *p* < 0.001, effect size = 0.777). Significant reductions were also observed in social triggers (mean difference = −8.427, *p* < 0.001, effect size = 0.626), situational triggers (mean difference = −9.715, *p* < 0.001, effect size = 0.491), thinking-related triggers (mean difference = −10.858, *p* < 0.001, effect size = 0.676), and physiological triggers (mean difference = −11.750, *p* < 0.001, effect size = 0.723). Detailed crude and adjusted comparisons are presented in Table 2.

**Table 2:** Crude and covariate-adjusted effects of the intervention on eating trigger scores.

Outcome	Post-intervention mean (intervention (*n* = 64) vs. control (*n* = 64))	Crude *p*-value	Adjusted mean difference (95% CI)	Adjusted *p*-value	Effect size
Emotional triggers	6.53 vs. 23.08	< 0.001	−14.83 (−18.60–−11.06)	< 0.001	0.777
Social triggers	9.66 vs. 21.53	< 0.001	−8.43 (−11.77–−5.09)	< 0.001	0.626
Situational triggers	13.61 vs. 25.05	< 0.001	9.72 (6.16–13.28)	< 0.001	0.491
Thinking triggers	7.97 vs. 21.41	< 0.001	−10.86 (−14.71–−7.01)	< 0.001	0.676
Physiological triggers	7.28 vs. 21.36	< 0.001	−11.75 (−15.39–−8.11)	< 0.001	0.723
Total score	9.66 vs. 21.53	< 0.001	−11.89 (−15.07–−8.71)	< 0.001	0.469

Crude between-group comparisons were performed using independent sample t-tests based on post-intervention scores. Adjusted mean differences and 95% confidence intervals were estimated using analysis of covariance (ANCOVA) controlling for marital status, education level, job status, residency, and baseline eating trigger scores. Effect sizes are reported as partial eta squared (η^2^).

In the intervention group before the study, 53.1% (*n* = 34) of participants had obesity, 35.9% (*n* = 23) were very obese, and 10.9% (*n* = 7) had morbid obesity, but after the intervention, it changed to 3.1% (*n* = 2) with ideal weight, 65.6% (*n* = 42) overweight, 25.0% (*n* = 16) obese, 4.7% (*n* = 3) very obese, and 1.6% (*n* = 1) with morbid obesity. In the control group before the study, 39.1% (*n* = 25) of the participants were obese, 40.6% (*n* = 26) were very obese, and 20.3% (*n* = 13) had morbid obesity but after the study it changed to 1.6% (*n* = 1) with ideal weight, 31.3% (*n* = 20) obese, 40.6% (*n* = 26) very obese, and 26.6% (*n* = 17) with morbid obesity. The BMI was not different between the two groups before the study, while it was significantly lower in the intervention group compared with the control group after the intervention (Table 3). The ANCOVA was used to control the effect of confounder variables (i.e., marital status, education level, job, living place, and BMI before the study). The results showed the BMI decreased significantly in the intervention group compared with the control group (Mean difference = −8.005, *P* < 0.001), effect size = 0.713.

**Table 3:** Comparison of participants' body mass index between the intervention and control groups.

Variable	Group	Before (mean ±SD)	After (mean ±SD)	Paired *t*-test	*p*-value
Body mass index	Intervention (*n* = 64)	35.74 ± 4.26	28.52 ± 3.56	−16.203	< 0.001
	Control (*n* = 64)	36.61 ± 3.50	36.57 ± 3.67	−0.195	0.846
Between-group comparison (independent sample *t*-test)					
**Time**		***t*** **-value**		***p*** **-value**	
Before intervention		1.273		0.205	
After intervention		13.559		< 0.001	

SD, standard deviation.

Independent sample t-tests were used to compare body mass index between the intervention and control groups at baseline and after the intervention. Paired sample t-tests were used to assess within-group changes in body mass index from baseline to post-intervention. Normality testing confirmed that all continuous variables met the assumptions for parametric analysis.

## 4 Discussion

This study aimed to evaluate the effectiveness of a nurse-led intervention on eating triggers for weight control. The findings demonstrated that, following the nurse-led educational intervention, the total score for eating triggers and all its dimensions significantly decreased in the intervention group compared to the control group.

The significant reduction in the total score of eating stimuli and its dimensions in the intervention group compared to the control group can be attributed to several key factors. Firstly, the educational intervention likely increased participants' awareness and knowledge about how various stimuli affect eating behaviors. With heightened awareness, participants may have become more conscious of their eating habits and triggers, contributing to a reduced influence of these stimuli. Additionally, the intervention provided tools and strategies for managing responses to eating cues, such as mindful eating, portion control, and understanding the role of emotional and environmental factors. These skills align with behavioral theories, such as the Theory of Planned Behavior, which emphasizes that knowledge and skills can lead to positive behavior changes (26, 27).

To the best of the authors' knowledge, this study is among the first to examine the impact of nurse-led interventions on eating motivation for weight control. As a result, direct comparisons with other studies are not feasible. Although limited evidence exists regarding nurse-led interventions specifically targeting eating triggers, several components of the present intervention have been examined in previous research. Behavioral therapy has consistently demonstrated effectiveness in modifying maladaptive eating behaviors by improving self-monitoring, cognitive restructuring, and coping strategies in individuals with obesity (28). Similarly, mindfulness-based approaches have been shown to improve awareness of hunger and satiety cues while reducing impulsive and emotionally driven eating behaviors (29). Emotion regulation strategies have also been associated with reductions in emotional eating and improved long-term weight control outcomes (30). Integrating these evidence-based strategies within a nurse-led framework may enhance accessibility and continuity of behavioral weight management interventions.

Eating triggers represent a multidimensional construct involving emotional, social, situational, cognitive, and physiological influences on eating behavior. The present study demonstrated that addressing these triggers through individualized nurse-led interventions resulted in significant improvements across all trigger domains. These findings suggest that eating triggers may serve as an important behavioral target for weight management interventions, as maladaptive responses to internal and external cues often contribute to overeating and weight gain. By focusing on trigger recognition and coping skill development, nurse-led interventions may provide participants with practical tools to manage real-life eating challenges and sustain behavioral changes.

Furthermore, the significant reduction observed in emotional eating triggers highlights the role of psychological factors in weight management. Emotional distress, social pressure, environmental cues, and cognitive distortions have been widely recognized as contributors to problematic eating behaviors. Addressing these factors through structured nurse-led support may help participants develop adaptive coping strategies and improve self-efficacy in managing eating behaviors.

Another important finding of the present study is that the nurse-led educational intervention effectively guided participants in the intervention group toward achieving weight loss and a healthier weight distribution. This intervention led to a notable reduction in BMI in the intervention group compared to the control group, underscoring its potential to support participants in reaching weight loss goals and improving overall weight classification.

These findings align with existing literature showing that structured, healthcare-professional-led weight management programs can significantly improve metabolic health outcomes (31). For instance, a randomized controlled trial (RCT) found significant weight loss in the nurse-led intervention group compared to the control group, underscoring the effectiveness of these interventions in weight management (32).

Several studies emphasize the impact of educational components within these programs. Sarver et al. observed differences in weight loss between participants receiving nutrition education and those who did not, though this difference was not statistically significant (33). However, another study reported that participants who received comprehensive education on nutrition and lifestyle changes experienced higher weight loss up to 12 months post-surgery compared with those lacking similar education (34).

Improvements in clinical measures, such as body weight and BMI, further validate the effectiveness of nurse-led interventions in achieving positive health outcomes (35). For example, through nurse-led strategies, Krishnasamy et al. demonstrated significant reductions in both weight and BMI (36). Similarly, a study involving intensive lifestyle intervention showed a ≥10% body weight loss within the first year (37), and a northern India study reported significant BMI reductions in participants following a nurse-led lifestyle modification intervention compared to the control group (2).

Moreover, a systematic review of 20 studies examining nurse-led weight management interventions found that three of nine studies with long-term follow-up (12 months to 2 years) reported significant reductions in weight or BMI between intervention and control groups (38).

Further systematic reviews support the efficacy of nurse-led diet and exercise interventions in preventing overweight and obesity across various age groups, with a notable impact on BMI reduction (23, 39, 40). Additionally, García-Rodríguez et al. demonstrated substantial weight, waist circumference, and BMI reductions and improved adherence to a Mediterranean diet and physical activity following a nurse-led intervention (41).

Contrastingly, Niu et al. (2021) found no significant difference in BMI between participants receiving a nurse-led web-based intervention and those in a web-based intervention without nurse involvement for type 2 diabetes management (42). The discrepancy may be due to the fact that the present study used an in-person intervention, unlike this study, which employed technology-based or multidisciplinary approaches. The delivery method (e.g., in-person vs. web-based) can significantly impact participant engagement and adherence.

Several limitations of this study should be considered when interpreting the findings. First, although adjustments were made for several demographic and baseline variables, other potential confounding factors such as dietary intake, physical activity levels, and use of weight-related medications were not fully controlled. Variations in participants' lifestyle behaviors or pharmacological treatments may have influenced weight outcomes and eating trigger responses, potentially affecting the observed intervention effects. Second, the study employed a non-randomized design, which may introduce selection bias and limit the ability to establish causal relationships between the intervention and study outcomes. Although statistical adjustments were performed to reduce the impact of confounding variables, residual confounding cannot be completely excluded. Third, the reliance on self-reported questionnaire data for assessing eating triggers may have introduced response bias or social desirability bias, potentially affecting the accuracy of participants' reported behaviors.

Additionally, the relatively short follow-up period limits conclusions regarding the long-term sustainability of behavioral changes and weight management outcomes. Future studies using randomized controlled designs, longer follow-up periods, and objective measurements of lifestyle behaviors are recommended to strengthen the evidence base. Another limitation of this study is the predominance of male participants in the sample. This gender imbalance may limit the generalizability of the findings, as obesity prevalence, eating behaviors, and responses to behavioral interventions can differ between men and women. Therefore, the findings of the present study may not fully represent obesity-related behavioral dynamics across both sexes. Finally, there is limited national research evidence supporting the present study findings.

## 5 Conclusion

This study highlights the effectiveness of a nurse-led intervention in addressing eating triggers, aiding weight control, and improving BMI. By fostering greater awareness of eating cues and equipping participants with practical strategies for healthier eating behaviors, the intervention significantly supported positive changes in eating habits and overall health. These results underscore the critical role of education and personalized support in nurse-led programs for sustainable weight management and improvements in metabolic health.

Future research can assess the long-term impact of nurse-led interventions on maintaining weight loss and managing eating triggers, with extended follow-up periods to evaluate sustainability.

## Data Availability

The original contributions presented in the study are included in the article/supplementary material, further inquiries can be directed to the corresponding author.
